# Supplementary material for: Satiation state-dependent dopaminergic control of foraging in Drosophila
Source: Sci Rep. 2018 Apr 10;8:5777. doi: 10.1038/s41598-018-24217-1 (PMC5893590; doi:10.1038/s41598-018-24217-1)
Supplement: Supplementary file 1 — Supplementary Information [file 41598_2018_24217_MOESM1_ESM.pdf]

Supplementary Information

Satiation state-dependent dopaminergic control of foraging in Drosophila

Dan Landayan[1#], David S. Feldman[2#], Fred W. Wolf[1,2*]

Supplementary Figures

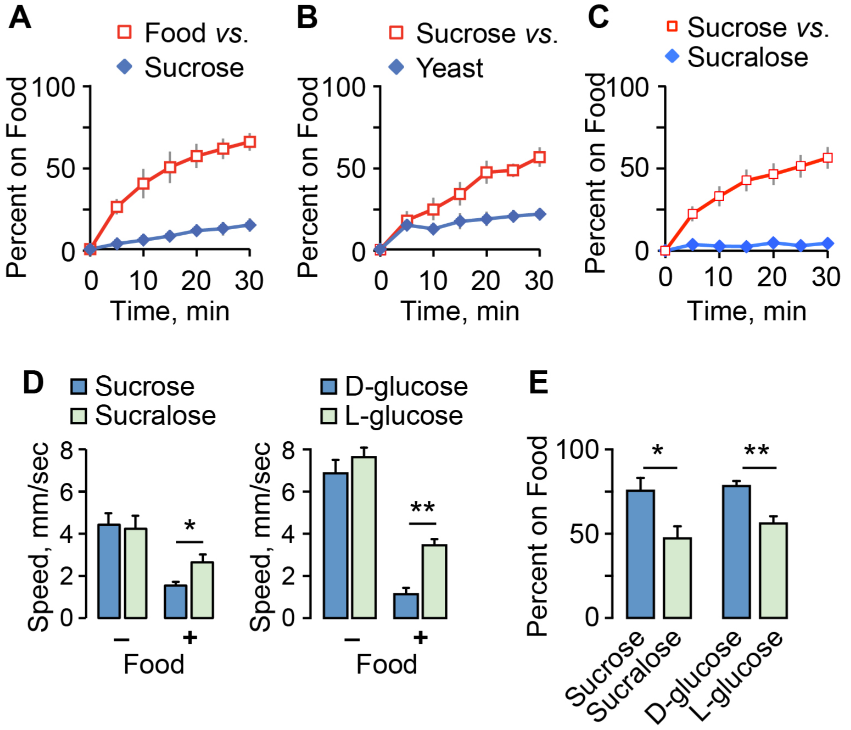

Figure S1, related to Figure 2. Effect of food composition on foraging behavior. **A**-**C**. Two-choice tests, where flies are presented with two sources that are directly adjacent in the arena center. Percent of flies occupying **A**. food *vs*. sucrose, **B**. sucrose *vs*. yeast, and **C**. sucrose *vs*. sucralose. **D**,**E**. Presentation of flies with a single source in the arena center. **D**. Locomotor speed of flies before and after addition of the indicated food source. t-test, **P<0.01, *P<0.05. n=6-10 groups. **E**. Food occupancy for the indicated food source. **P<0.01, *P<0.05. n=8 groups.

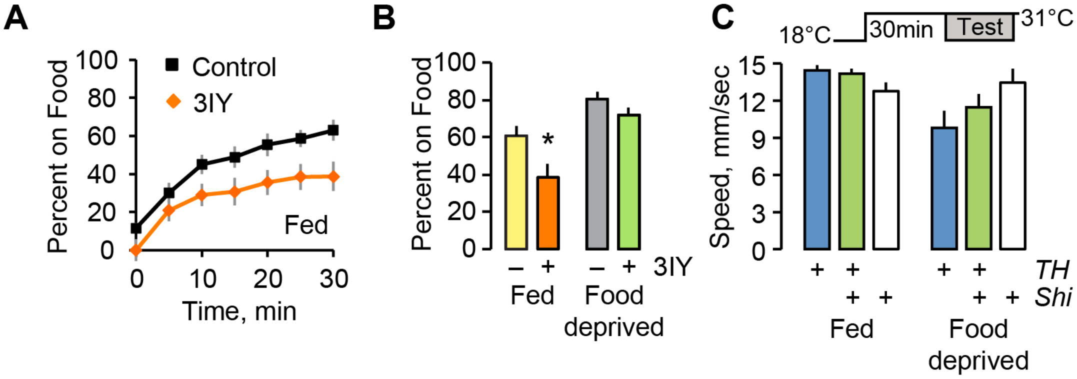

Figure S2, related to Figure 3. Food occupancy after treatment with 10 mg/mL 3-iodotyrosine (3IY). **A**. Time course. **B**. Percent occupancy. t-test, *P<0.05. n=8 groups. **C**. Locomotor speed with acute inactivation of *TH-Gal4* neurons. n=8-11 groups.

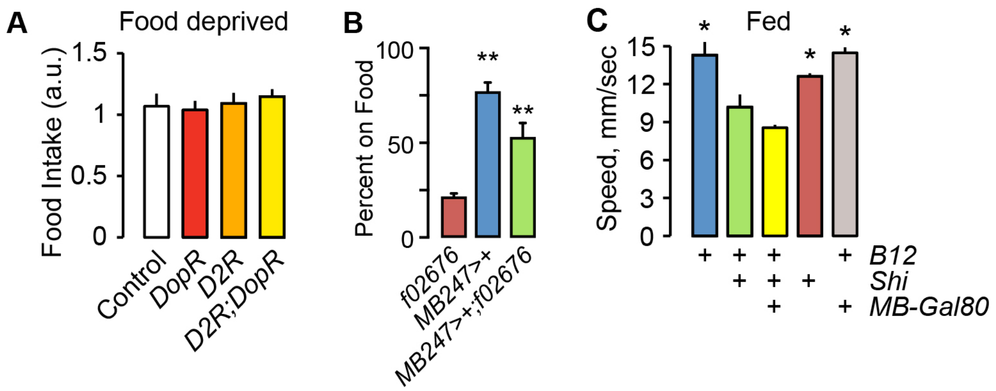

Figure S3, related to Figure 4. Dopamine receptor neuron manipulation. **A**. Food intake for the indicated genotypes. n=10 groups. **B**. Food occupancy for genetic rescue restricted to the mushroom bodies. P<0001, One-way ANOVA/Tukey's. n=12 groups. **C**. Locomotor speed in fed flies of the indicated genotypes. P<0.0001 One-way ANOVA/Bonferroni compared to *B12-Gal4>UAS-Shi^{ts}*. n=8-12 groups.
